# Beard & Rockwell on Medical and Surgical Use of Electricity

**Published:** 1875-02

**Authors:** 


### Bibliographical.

A PRACTICAL TREATISE on the Medical and Surgical uses of Electricity. Including: Localized and general Faradization; Localized and central Galvanization; Electrolysis and Galvano-Cautery. By George M. Beard, A.M., M.D., member of the New York Society of Neurology and Electrology, etc., and A. S. Rockwell, A.M., M.D., member of the New York Society of Neurology and Electrology, etc. Second edition, revised, enlarged, and mostly re-written. With nearly two hundred illustrations. New York: William Wood & Co., 27 Great Jones street. 1875. One volume, octavo, cloth, pp. 794. Price $6.25.

The question is often asked us, "What *real progress* has faradization and electrolysis, in the hands of the specialists, made towards the substantial cure of disease?" Others desire to know if any definite therapeutical principles for the government of electrization in disease have been established; to what diseases is this method of treatment applicable; what measure of beneficial results can be relied upon; what is the applicability of the different currents; how and where and when to be used; what may we promise the patient in any given case, etc. It is one of the objects of the work of Drs. Beard and Rockwell to answer such questions, and the fact that we have before us a new edition, revised, enlarged, and mostly re-written, argues that the spirit of inquiry which is manifested by our readers and friends is more or less general among medical men throughout the country, and leads us to believe that our authors are responding in a satisfactory way to this general thirst for knowledge.

We know not how we can better bring the scope of the new edition before our readers, in the space at our disposal, than by the introduction of a few extracts from the preface of the new work: "The use of general faradization as a constitutional tonic in a wide variety of affections is now well established, and the effects that we have claimed for it have been confirmed in full detail by competent observers at home and abroad. * * * The section on Electro-physics is much enlarged. Observation has convinced us that the one great defect in those who practice electro-therapeutics is ignorance of the physical relations of electricity. * * * The chemistry of the batteries is explained in full detail. * * * To Ohm's Law, at once so important and so difficult, a separate and special chapter has been assigned; and no effort has been spared to make it clear in all its practical relations to all trained minds who will give it close and careful attention. * * * The method of central galvanization that we have systematized and introduced to the profession since the publication of the first edition is here described and illustrated in full detail. * * * In the chapter on apparatus we have endeavored to represent with fairness and impartiality the best workmanship and the most recent improvements. * * * A new chapter on General Suggestions has been added, in which the attempt has been made to answer in detail the various practical queries that so annoy the beginner. * * * In the clinical part of electro-medicine a number of entirely new chapters have been added, and all of the chapters have been recast. The number of cases has been increased nearly twofold, the failures and successes being fairly represented. * * * We may call especial attention to the chapters on Diseases of the Skin, wherein, besides many other cases, are detailed the remarkable results of central galvanization in chronic eczema and prurigo, and to the chapter on Diseases of Childhood, in which are recorded the results of experiments in the treatment of whooping-cough, marasmus, and debility, and also the fact of the remarkable tolerance of childhood to electricity."

Whilst acknowledging ourselves incompetent to properly criticize the work of Beard and Rockwell, we are safe in presenting it to our readers as the standard authority upon the specialty to which it is devoted, in America.

